# The matrix (M) protein of newcastle disease virus binds to human bax through its BH3 domain

**DOI:** 10.1186/1743-422X-8-385

**Published:** 2011-08-03

**Authors:** Aidin Molouki, Yi-Te Hsu, Fatemeh Jahanshiri, Syahril Abdullah, Rozita Rosli, Khatijah Yusoff

**Affiliations:** 1 Department of Microbiology, Faculty of Biotechnology and Biomolecular Sciences, Universiti Putra Malaysia, 43400 UPM, Serdang, Selangor DE, Malaysia; 2 Department of Biochemistry and Molecular Biology, Medical University of South Carolina, Charleston, SC, USA; 3 Department of Obstetrics and Gynaecology, Faculty of Medicine and Health Sciences, Universiti Putra Malaysia, 43400 UPM, Serdang, Selangor DE, Malaysia; 4 Institute of Biosciences, Universiti Putra Malaysia, 43400, Serdang, Selangor DE, Malaysia

**Keywords:** NDV, AF2240, Apoptosis, Matrix protein, Fusion protein, Bcl-2 homology domain, Protein-protein interaction, HeLa

## Abstract

The underlying mechanisms by which Newcastle disease virus (NDV) kills cancer cells are still unclear. Recent discoveries have shown that many viruses contain Bcl-2 homology-like domains which enabled their interaction with Bcl-2 family members, and thereby accounting for their virulence and pathogenicity. Alignment of the protein sequences of Malaysian strain of NDV, known as AF2240, with those from members of the human Bcl-2 family showed many similar regions; most notably we found that its matrix (AF2240-M) protein, large (AF2240-L) protein and fusion (AF2240-F) protein all contain BH3-like regions. In addition, there are BH1-like domains in these proteins, where AF2240-F and Mcl-1 share 55% identity within this region. To further investigate our hypothesis that the presence of the BH3-like domains in these proteins may convey cytotoxicity, AF2240-M and AF2240-F genes were cloned into pFLAG and pEGFP.N2 vectors and transfected into HeLa cells. The expression of these constructs promoted cell death. As shown by flow cytometry, AF2240-M protein with deleted BH3-like region showed five-fold decrease in apoptosis. Moreover, the construct containing the N-terminal of AF2240-M showed nearly the same cell death rate as to that of the full-length protein, strongly suggesting that the BH3-like domain within this protein participates in promoting cell death. Moreover, AF2240-M transfection promoted Bax redistribution to mitochondria. Therefore, to determine whether there is any direct interaction between NDV viral proteins with some members of the Bcl-2 family, various constructs were co-transfected into HeLa cells. Co-immunoprecipitation trials showed that the AF2240-M indeed directly interacted with Bax protein via its BH3-domain, as the mutant proteins failed to interact with Bax. AF2240-F failed to interact with any of the tested proteins, although Bcl-XL slowed down the rate of cell death caused by this construct by nearly five-fold. In a parallel experiment, the level of expression of endogenous Bax and Bcl-2 after infection of HeLa cells with NDV was assessed by qRT-PCR, but no statistically significant change was observed. Consequently, the Bax/Bcl-2 ratio at the mRNA level did not alter. Overall, our study has shed additional light into the mechanisms by which NDV induces apoptosis.

## Introduction

Newcastle disease virus (NDV), an avian paramyxovirus, affects most species of birds and is considered one of the most devastating viruses that can cause great economic damage to the poultry industry [1]. Its many strains have been mainly categorised by its pathotype and clinical effects rather than by its structure. Strain AF2240 is a viscerotropic velogenic strain of NDV that causes a high mortality rate and was isolated during an outbreak in the country in the 1960s. It is now used as the challenge virus in vaccine trials in Malaysia [2].

NDV belongs to the genus *Avulavirus *from the family of *Paramyxoviridae *[3]. It has a non-segmented single-stranded negative-sense RNA genome of nearly 15 kb [4-6]. The virion is enveloped with a lipid bilayer membrane derived from the host cell membrane [7]. This structure consists of 6 proteins. HN and F fusion glycoproteins are embedded in the lipid membrane. The matrix (M) protein is associated with the inner surface of the membrane. Inside the virion, three proteins, nucleocapsid (NP), phosphoprotein (P), and large (L) protein, constitute the viral transcriptase complex. The interaction between these macromolecules and their binding domains have yet to be defined completely [7]. Upon entry into the host cell, NDV genome is subjected to two separate mechanisms of transcription and replication that result in the production of more viruses.

The F glycoprotein mediates virus fusion with the host cellular membrane. Its gene has 1791 bp with an open reading frame of 1662 bp and it codes for F0 precursor with 553 amino acids. The precursor is then cleaved at residues 116-117 to generate F1 and F2 disulphide-linked polypeptides [8,9]. The transmembrane domain of F is located at the C-terminal region of F1. Three hapted repeat sites have been reported that are strain specific [10] and also six glycosylation sites have been previously identified [11].

The M gene of strain AF2240 has 1223 bp with an ORF of 1093 bp. Its translated product contains 364 amino acids for all reported strains and has a molecular weight of approximately 40 kDa [11]. It is relatively hydrophobic and non-glycosylated with many basic residues. It has been reported that M is associated with the N-terminal segment of HN protein located in its inner surface [12]. It is also believed that it interacts with the NP protein, but the exact binding domains are yet to be determined [7]. The nucleocapsid itself is a herringbone-like structure comprising thousands of NP subunits that are associated tightly with several copies of P and L proteins.

NDV appears to selectively kill cancer cells while sparing normal human cells [13]. It is believed that NDV kills avian cells and human cancer cells by apoptosis [14-16]. On the other hand, cell death through apoptosis is initiated via either intrinsic or extrinsic pathway [17]. The Bcl-2 family of proteins plays an important role in the regulation of mitochondrial checkpoints that are involved in both intrinsic and extrinsic apoptotic signaling pathways [18]. These proteins display both pro- and anti-apoptotic functions [19] and have one or more of the conserved domains known as the Bcl-2 homology (BH) domains 1 to 4. These family members can interact with each other or other proteins via these conserved domains.

Numerous publications have demonstrated the anti- or pro-apoptotic effects of viruses and their respective viral proteins in eukaryotic cells [20], but it is only recently that researchers have begun to investigate the sequence similarity between viral proteins and cellular apoptotic proteins. Since the late 20th century, it was shown that some viruses contain protein homologs to Bcl-2 family members and that many of these domains enable these proteins to interact with Bcl-2 family members [21-29]. These studies provide an initial clue on the underlying mechanisms behind the apoptosis regulating effect of these viruses.

Here, we report for the first time that NDV viral proteins contain BH domain-like regions, and that AF2240-M and AF2240-F display pro-apoptotic effect in HeLa cells. Previously, it was shown that strain AF2240 has a different HN protein compared to that of other strains [30]. However, now we show that there are additional unique features within the BH1- and BH3-like regions in M, F and L proteins of strain AF2240, and that AF2240-M interacts with human Bax protein by its BH3 domain.

## Materials and methods

### Cell culture

Human cervical cancer cell line, HeLa, was purchased from American Type Culture Collection (ATCC) and cultured in Dulbecco's modified Eagle's medium (DMEM) supplemented with 10% fetal bovine serum (FBS), 100 U of penicillin/ml and 100 μg of streptomycin/ml in 5% CO_2 _at 37°C, and routinely checked for mycoplasma contamination. The cells were infected with NDV as previously described [15].

### qRT-PCR

Real-Time RT-PCR was performed with a two-step system optimized for use with Rotor-Gene 6000 Real-Time PCR machine (Corbett, Australia). Briefly, at different time points post-infection, HeLa cells were harvested and total cellular RNA was isolated using RNeasy Mini kit (Qiagen, USA). cDNA was prepared with a QuantiTect^® ^RT system (Qiagen, USA) according to manufacturer's instructions. Next, normalized cDNAs were used in a Hotstart qPCR with Rotor-Gene SYBR green kit (Qiagen, USA) together with Bax and Bcl-2 QuantiTect primer sets (Qiagen, USA). The cycling conditions started with initial 5 min at 95°C followed by 35-40 two-step cycles of 5 sec at 95°C and 10 sec at 60°C. A melting curve analysis was performed in the end of the reaction to verify the specificity and identity of the PCR products. Finally, the ΔΔCt [= (Ct of target gene in sample - Ct of housekeeping gene in sample) - (Ct of target gene in control - Ct of housekeeping gene in control)] and expression (2^-ΔΔCt^) values were calculated.

### Protein sequence analysis

All protein sequence alignments were performed with SIM+LALNVIEW at ExPASy proteomics server http://192.33.215.47/tools/sim-prot.html. Protein BLAST (BL2SEQ) was used to identify sequence similarities between NDV viral proteins and human cellular proteins. Prediction of transmembrane helix and intervening loop region was done by TMHMM software, and prediction of secondary structures was done by PELE software, both of which are located at San Diego supercomputer center server http://workbench.sdsc.edu/.

### Plasmid constructs

Total RNA was extracted with QIAamp Viral RNA Mini Kit (Qiagen, USA) from pure stock of Malaysian strain of NDV strain AF2240, which was grown in 9-day old embryonated chicken eggs and purified as previously described [31]. cDNA was made with RevertAid kit (Fermentas, USA). Specific primers were used for amplifying the cDNAs to enable tagging and cloning of AF2240-M and AF2240-F viral genes. The PCR primers used for subcloning the AF2240-M gene into pFLAG-CMV-5a vector (Mammalian carboxy-terminal FLAG transient expression kit, Sigma, USA) were 5'-CTTG*GAATTC***ACC**ATGGACTCATCCAGGGCAATC-3' for forward and 5'-CTTG*GTCGAC*TTTTTTGAAAGGGTTGTATTTAGC-3' for reverse. The pFLAG-M-N-terminal mutant was generated by the same forward primer but with a different reverse primer, 5'-CTTG*GTCGAC*TTGTGGGGTGATTTGCTTCTT-3' to amplify 40 a.a. of the N-terminal. The AF2240-F gene primers used for PCR amplification and subcloning into the same vector were 5'-CTTG*GAATTC***ACC**ATGGGCTCCAAGTCTTCTACC-3' for forward and 5'-CTTG*GTCGAC*TGTTCTTGTGGTGGCTCTCAT-3' for reverse. Restriction endonuclease sites *EcoR*I and *Sal*I were chosen for subcloning the PCR fragments into the MCS site of the pFLAG vector. The AF2240-M gene PCR primers used for subcloning into pEGFP.N2 (Clontech, USA) were 5'-AGCTCA*AGATCT***ACC**ATGGACTCATCCAGGGCA-3' for forward and 5'-GAGGTC*AAGCTT***GC**GAAAGGGTTGTATTTAGCA-3' for reverse (restriction endonuclease sites *Bgl*II and *Hin*dIII) having two extra nucleotides (in bold) to clone in the same reading frame of the EGFP tag. Also, a Kozak's ACC sequence (in bold) was included in all forward primers to promote the translation of the target genes in eukaryotic cells. PCR was carried out with a recombinant *pfu *DNA polymerase (Fermentas, USA). All PCR products were gel purified and were first cloned into pJET vector (CloneJET PCR cloning kit, Fermentas, USA) to increase PCR product quantity and enhance the chance of cloning to the main target vectors. Generation of pGFP-Bax, pGFP-Bad and pGFP-Bcl-XL has been described previously [32]. pcDNA3-FLAG-BimL was a kind gift from Professor Roger Davis, MIT Medical School, Boston, MA, USA. All plasmids were transformed into TOP10 *E. coli*, grown in LB broth and extracted with PureLink kit from Invitrogen.

### Generation of AF2240-M-ΔBH3 deletion mutant

The plasmid pFLAG-M-ΔBH3 was produced by deletion of the BH3-like domain in AF2240-M gene. Briefly, two primers flanking the region between base pairs 67-111 were designed in a way to not disturb the FLAG-tag reading frame (Forward: 5'-ACCCCACAATACAGGATCCAG-3' and Reverse: 5'-TGCTAATAGGCTGCTGGAGGGAAG-3'). PCR was run for 20 cycles with 50 ng of pFLAG-M as template. Recombinant *pfu *DNA polymerase was used to proof-read the entire product. Later, the PCR product was subjected to digestion with *Dpn*1 enzyme to eliminate the methylated template DNA. After gel purification, the linear PCR product was ligated with T4 ligase for 3 h at RT, and then transformed into TOP10 *E. coli*.

### Co-transfection and fluorescent microscopy

For transfection, HeLa cells were cultured in 10 cm in diameter plates at 2 × 10^5 ^cells/plate. The next day, at a confluency of 70-80%, the medium was removed and cells were washed with PBS. Transfection or co-transfection of cloned plasmids were done with Lipofectamine LTX and PLUS reagent (Invitrogen, USA) according to manufacturer's instructions. Cells were analyzed between 12 h to 48 h post-transfection by flow cytometery, co-immunoprecipitation, light and fluorescent microscopy.

### Flow cytometry and PI staining

On the day of experiment, cells were detached with Accutase (PAA, USA) and washed with cold PBS (pH 7.4). Floating cells in medium which had died after transfection were also collected. The pellets were resuspended in ice-cold absolute ethanol to fix cells. Cells were kept in -20°C freezer until they were pelleted down at the time of experiment. Three hundred microliters of Propidium iodide (1 μg/ml) containing 10 μg/ml DNase-free RNase A in PBS was added to each fixed cell pellet. The pellets were resuspended and incubated for 10 min before addition of a same volume of sheath buffer. The stained cells were then analyzed with BD FACSCanto II flow cytometer. The data were analysed with BD FACSDiva and Cyflogic softwares.

### Subcellular fractionation

Subcellular fractionation was performed as previously described [15]. Briefly, transfected cells were collected at different time points and resuspended in mitochondrial buffer (70 mM Tris-HCl, 0.25 M sucrose and 1 mM EDTA, pH 7.4). An equal volume of ice-cold digitonin lysis buffer (2 mg/ml, 19.8 mM EDTA, 0.25 M D-mannitol and 19.8 mM MOPS, pH 7.4) was added for 90 s. Samples were then centrifuged twice at 300 g for 5 min to pellet the nuclei. The supernatant was further centrifuged at 17,000 g for 20 min to separate mitochondria from the cytosol. The supernatant was then subjected to SDS-PAGE and Western blotting with mouse monoclonal anti-Bax 2D2 antibody (Invitrogen, USA).

### Co-immunoprecipitation

In order to study protein-protein interactions, the co-immunoprecipitation approach was tried. A FLAG kit (Sigma, USA) was used in all Co-IP trials. Briefly, all transfected cells were harvested and washed with PBS twice before being resuspended in 100 μL of 2% CHAPS lysis buffer (2% CHAPS, 10 mM HEPES pH 7.4, 150 mM NaCl, and 1 tablet of Roche cOmplete™ protease inhibitor) [33,34]. After quantification with Bradford method, the normalized cell lysates were added to 0.5 mL microtubes containing 40 μL of anti-FLAG M2 affinity gel. For positive control, 200 ng of FLAG-BAP™ fusion protein was used. The microtubes were rotated end-to-end at 4°C overnight. After incubation, the microtubes were centrifuged at 8000 g for 30 sec. The supernatants were removed and the resins were washed twice with 0.5% CHAPS buffer. The cleaned resins were then resuspended in 30 μL of SDS-PAGE sample buffer and boiled for 10 min. The microtubes were then centrifuged at maximum speed for 5 min and the supernatants were used in SDS-PAGE and Western blotting.

### SDS-PAGE and Western blotting

Proteins in the SDS-polyacrylamide gel were electrotransferred onto Immobilon-P membrane (Millipore Corp., USA) and after transferring, the membrane was blocked with a casein buffer (Pierce, USA). The membrane was incubated either with a monoclonal anti-FLAG clone M2 raised in mouse (Sigma, USA), or polyclonal anti-GFP ab290 raised in rabbit (Abcam, USA) at a dilution of 1:1000 overnight at 4°C. The blot was then respectively incubated with an AP-conjugated goat anti-mouse (Bethyl, USA), or AP-conjugated goat anti-rabbit (Sigma, USA) at a dilution of 1:1000 for 1 h at room temperature. NBT/BCIP alkaline phosphatase buffer (100 mM Tris-HCl pH 9.5, 100 mM NaCl, 10 mM MgCl2, containing 3 mg/ml NBT and 1.5 mg/ml BCIP) was used to develop the blotted membrane (Fermentas, USA).

### Statistical analyses

All statistical analyses were performed by Microsoft Excel. All error bars indicate the standard error of the mean (SEM). p-values less than 0.05 (*), 0.01 (**) and 0.001 (***) reject the null hypothesis and are referred as statistically significant.

## Results

### Infection of HeLa cells with NDV did not alter Bax and Bcl-2 mRNA levels

**Figure 1:**
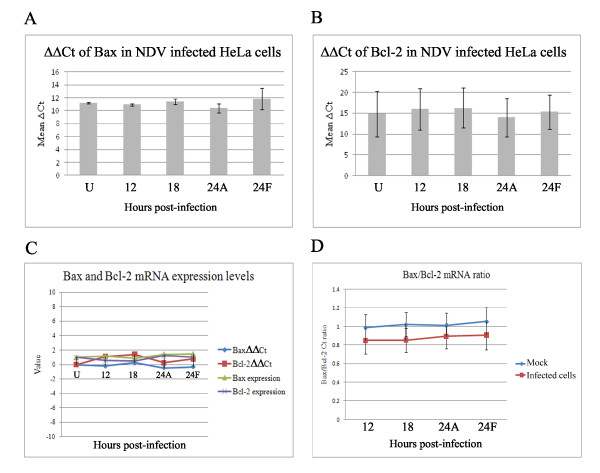
**Real-Time PCR results showing expression of Bax and Bcl-2 mRNAs in HeLa cells following infection with NDV**. **A**. ΔCt of Bax mRNA. **B**. ΔCt of Bcl-2 mRNA. **C**. Bax and Bcl-2 mRNA expression levels. **D**. Ratio of Bax to Bcl-2 Ct values. Note: All values were against housekeeping gene 18s rRNA Ct values. No statistically significant difference versus uninfected control cells was observed. Error bars indicate standard error of the mean from three independent measurements. U: uninfected cells. A: adherent infected cells. F: floating infected cells.

HeLa cells were infected with NDV and their RNA was extracted at different time points post-infection. A relative comparison of the expression of endogenous Bax and Bcl-2 before and after NDV infection was studied. Real-Time PCR with the generated cDNA was carried out and ΔCt, ΔΔCt and expression (2^-ΔΔCt^) values were calculated according to the Cts of samples and controls (Figure 1). The values were compared to that of the housekeeping gene 18S rRNA. Surprisingly, the mRNA levels of Bax and Bcl-2 before and after NDV infection did not alter much as no notable increase or decrease was observed in the Ct values during multiple trials. Thus, the ratio between Bax and Bcl-2 did not show a statistically significant alteration following NDV infection (Figure 1D). At 24 h post-infection many cells died and detached. These cells were collected separately from adherent infected cells. For easier presentation, the uninfected cells, adherent infected cells and floating infected cells were marked as U, A and F, respectively.

### Multiple BH domain-like regions in NDV viral proteins

By BLASTing NDV viral protein sequences in the BL2SEQ online software at NCBI website, we found many similar regions between these six proteins (M, L, F, HN, NP, P) and human cellular proteins (Table 1). Surprisingly, AF2240-M protein scored the highest in terms of sequence homology to some mammalian proteins such as RAS p21 activator, B-cell transcription factor 4, and spectrin beta chain, compared to other viral proteins. AF2240-L and AF2240-F came in second and third respectively in the number of matching homologous sequences. On the other hand, AF2240-HN, AF2240-NP and AF2240-P did not show much similarity with mammalian proteins. Importantly, we found many similarities between NDV protein sequences and Bcl-2 family members, especially for AF2240-L (Table 1). To narrow down our alignment studies, we compared Bcl-2 homology domains (BH1, BH2, BH3 and BH4) of many Bcl-2 family members with sequences from NDV viral proteins by SIM+LALNVIEW online software at ExPASy website and identified multiple BH domain-like regions in NDV viral proteins.

**Table 1:** Homologous regions between NDV viral proteins and human cellular proteins.

Names	Sequences	Identity %	Score (Bits)	Gap %
AF2240-M B-cell transcription factor 4 leukemia	27 LQDTGDGKKQI 38 LQDTGDILQQI ****** **	72.7	36.0	0.0
AF2240-M Glucagon-like 1 receptor	297 QWDSLLSYSKCLS 213 QWDGLLSYQDSLS *** **** **	69.0	27.8	0.0
AF2240-M Cholecystokinin B receptor	128 VFSVVQA--PRVLQ 191 VYTVVQPVGPRVLQ * *** *****	64.0	26.9	14.0
AF2240-M Fibrous sheath-interacting 2	150 SVNAVNHVK 1506 SVNGGNHIK *** ** *	66.0	20.2	0.0
AF2240-M Spectrin beta chain, brain 4	46 LDSWTDS 2484 LDSWTDS *******	100.0	26.5	0.0
AF2240-M Cytochrome Oxidase	182 WDSLASFRKSLSP 298 WDSLLSYSKCLSP **** * * ***	69.2	49.0	0.0
AF2240-M RAS p21 protein activator	4 VLQDTGDGK 449 VLNDTVDGK ** ** ***	77.0	21.0	0.0
AF2240-M Bid	80 DNPGHEL 95 DALGHEL * ****	71.4	24.0	0.0
AF2240-M Bax	298 WDSLLSY 158 WDGLLSY ** ****	85.7	46.0	0.0
AF2240-L Bcl-2	1540 DFIEMSAKL 110 DFAEMSSQL ** *** *	66.7	31.0	0.0
AF2240-L Bid	19 NTSRSEE 147 NVSRSEE * *****	85.7	29.0	0.0
AF2240-L Bid	86 LRTYVR 2145 LRTYLR **** *	83.3	27.0	0.0
AF2240-L Bad	451 EFEPCIEYD 6 EFEPSEQED **** *	55.6	25.0	0.0
AF2240-F F-box protein FBW7	21 LILSCICL 8 LILSCICL ********	100.0	29.9	0.0

AF2240-M showed most matching sequences both to cellular proteins and also Bcl-2 family members compared to other viral proteins and strains.

**Figure 2:**
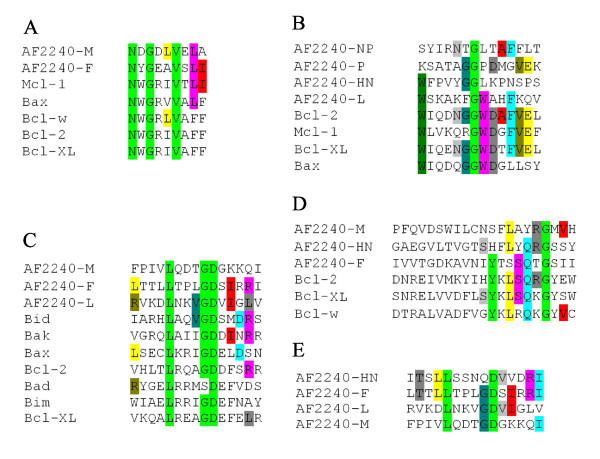
**A comparison between Bcl-2 homology domains of Bcl-2 family members and sequences from NDV strain AF2240 viral proteins**. Only identical residues between AF2240 proteins and Bcl-2 family members are highlighted. **A**. BH1 domain-like regions. Note that five out of nine residues of AF2240-F and Mcl-1 in this region are identical. Most conserved residues asparagine, glycine and valine are highlighted in green. **B**. BH2 domain-like regions. A few conserved amino acid residues observed. **C**. BH3 domain-like regions. Most conserved amino acid residues leucine, glycine and aspartic acid are highlighted in green. **D**. BH4 domain-like regions. Only AF2240-F shows four identical residues to a small segment of the BH4 domains. **E**. A comparison of BH3 domain-like regions between individual proteins of NDV strain AF2240 virus. Note that this domain is present in four different proteins. Conserved amino acid residues leucine, glycine and aspartic acid are present in three out of the four viral proteins (highlighted in green). AF2240-F and AF2240-HN share many similar residues but the conserved glycine residue is missing in AF2240-HN.

A highly similar BH1-like region was identified in AF2240-M matrix protein, with four out of eight residues being identical to those of Bax (Figure 2A). On the other hand, AF2240-F glycoprotein showed 55% BH1 similarity with Mcl-1 by having five out of nine residues identical. Some known conserved residues of BH1 such as asparagine, glycine, valine, and leucine are present in both AF2240-M and AF2240-F. BH1-like regions are also present in AF2240-HN and AF2240-L proteins, but with far lower scores, with at most four out of nine matching residues. Thus, they were not further considered due to the lack of conserved residues. Moreover, we found similar BH3-like regions in AF2240-L, AF2240-F, and AF2240-M proteins (Figure 2C). Within this domain, the amino acids leucine, glycine, and aspartic acid are highly conserved among various members of the Bcl-2 family and surprisingly, they are also present in all BH3-like regions in AF2240-L, AF2240-F and AF2240-M proteins. Our alignment studies did not reveal any considerable BH2- or BH4-like region in any of the NDV viral proteins. The closest sequences are shown in Figure 2B for BH2, and Figure 2D for BH4, but most conserved residues are missing.

Subsequently, the BH3-like regions of AF2240 proteins were aligned with each other to investigate their similarity (Figure 2E). The AF2240-HN glycoprotein, which showed low similarity to those of Bcl-2 family, surprisingly, had up to six matching residues with AF2240-F glycoprotein. Although this region is not entirely considered a BH3-like region, AF2240-HN only lacked one conserved residue, glycine.

### AF2240 shows unique features compared to other strains

**Figure 3:**
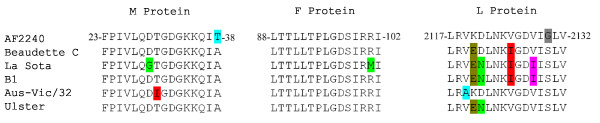
**An extended comparison of the BH3 domain-like regions between M, F, and L proteins of various strains of NDV**. Note the unique residue threonine 38 for AF2240-M. Also note one unique glycine 2130 residue for AF2240 L protein and some sequence variations between different L proteins.

All BH3-like sequences of AF2240 were compared to five other known NDV strains (Figure 3). The residues threonine 38 and glycine 2130, in the BH3-like domain of AF2240-M and AF2240-L respectively, are unique to the strain AF2240. Other strains show some shared residues which are not present in AF2240-L at all. AF2240-F does not show any uniqueness and most residues are similar to the other five strains. Only strain La Sota shows a different methionine residue in the BH3-like region of its F protein.

**Figure 4:**
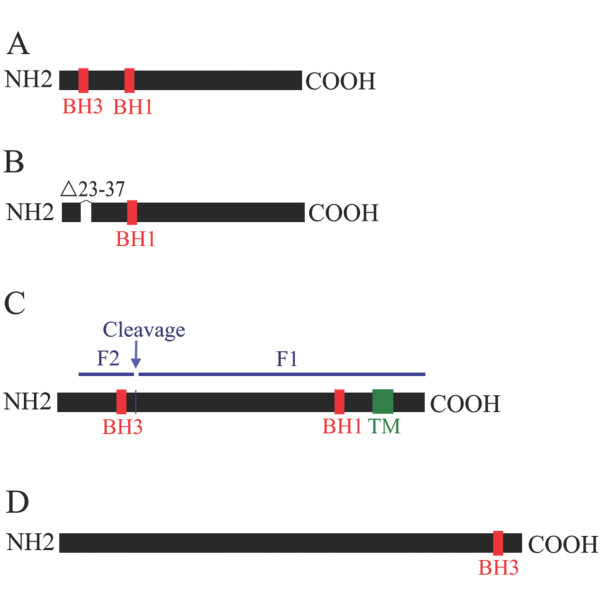
**Schematic diagrams showing the locations of various BH domain-like regions in different NDV viral proteins**. **A**. Location of BH1 and BH3 domain-like regions in AF2240-M protein. Both regions are located near the N-terminus. **B**. AF2240-M-ΔBH3 lacking a.a. 23-37. **C**. BH1 and BH3 domain-like regions in AF2240-F glycoprotein. The area shaded green represents the transmembrane segment. **D**. BH3 domain-like region in AF2240-L protein.

Both BH1- and BH3-like regions of AF2240-M matrix protein are located within the N-terminal segment of the protein (a.a. 99-107 and 27-32, respectively; Figure 4A). In addition, by using TMHMM software, we showed that the BH3-like region in M protein is located in the only area that exhibits a minor transmembrane binding ability in AF2240-M (data not shown). Later we generated the mutant AF2240-M-ΔBH3 that lacked the entire BH3-like region between amino acids 23-37 (Figure 4B), in order to study the effect on cell death and further analyze the region.

The BH3-like region on AF2240-F protein is located within F glycoprotein F2 sequence between a.a. 88 to 102 (Figure 4C), right before the cleavage site at a.a. 116-117. BH1-like region of AF2240-F is located in the C-terminal end between a.a. 411-419, located on F1 before the transmembrane domain at a.a. 501-527 [11]. This observation was further confirmed by TMHMM prediction software (data not shown). Both Bcl-2 homology domain-like regions on AF2240-F are not within any of the hapted repeats or glycosylation sites.

Compared to members of the Bcl-2 family, the Bax protein has the highest identity-hits with AF2240-M. Subsequently, we also found out that the Bax protein has the highest sequence homology with AF2240-M protein compared to other tested NDV strains. We also found a homologous sequence WDSLLSY (Table 1), with 85.7% identity to the α-8 helix sequence of Bax protein. This sequence is a part of the BH2 domain of Bax [35] and surprisingly, no other Bcl-2 family member shows any higher homology rate with Bax, than AF2240-M does.

The PELE software also showed that BH3-like regions of AF2240-L, AF2240-F and AF2240-M were all in random-coil regions and the BH3-like region of AF2240-F is right after a Beta-strand, but none of them are in helical areas, unlike those of many Bcl-2 family members.

### AF2240-M and AF2240-F transfections promote apoptosis

**Figure 5:**
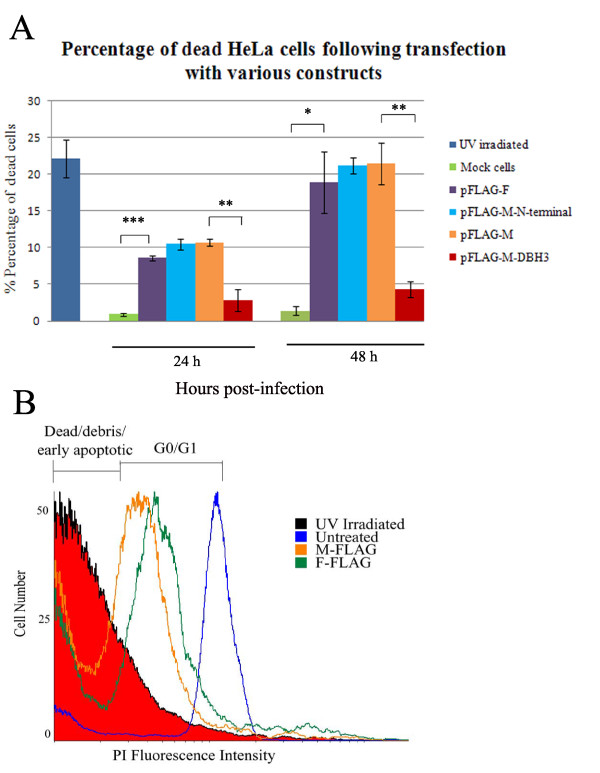
**Quantification of dead HeLa cells following transfection with various plasmid constructs**. **A**. Percentage of dead cells only, by flow cytometry and PI staining as a result of transfection with various constructs. I. pFLAG-M-N-terminal. II. pFLAG-M-ΔBH3. III. pFLAG-M. IV. pFLAG-F. V. UV-irradiated cells. **B**. A histogram analysis of flow cytometry data. The number of dead cells caused by pFLAG-M transfection was always slightly higher than those from pFLAG-F transfection. Note the G0/G1 region shift in cells transfected with pFLAG-M and pFLAG-F.

To assess the effect of M and F proteins in cell death regulation, AF2240-M and AF2240-F cDNAs were cloned into pFLAG-CMV-5a vector and transfected into HeLa cells. Cells viability was analysed by flow cytometry and fluorescence microscopy. We found that AF2240-M transfection caused rounding up of cells and membrane blebbing as early as 12 h post-transfection. Cell death was then confirmed by PI staining and analysis by flow cytometer at 24 h and 48 h post-transfection (Figure 5A).

To determine the role of the BH3 domain in NDV viral protein-induced cell death, we truncated the BH3 domain of M and transfected the resulting mutant construct into HeLa cells. As shown in Figure 5A, truncation of the BH3 domain resulted in a nearly five-fold decrease in cell death compared to the wild-type AF2240-M (from 21.49% down to 4.32%). In addition, we showed that transfection of the plasmid containing the N-terminal segment of AF2240-M (40 a.a.) also led to apoptosis, and the rate was almost comparable to the full-length AF2240-M (21.21%). pFLAG-F showed 12% less cell death compared to pFLAG-M and this was further evaluated by histogram analyses shown in Figure 5B. We observed a shift for the G0/G1 phase peak in the cells transfected with pFLAG-M and pFLAG-F compared to untransfected cells, although the reading threshold was kept constant for each sample (10,000 events/run).

**Figure 6:**
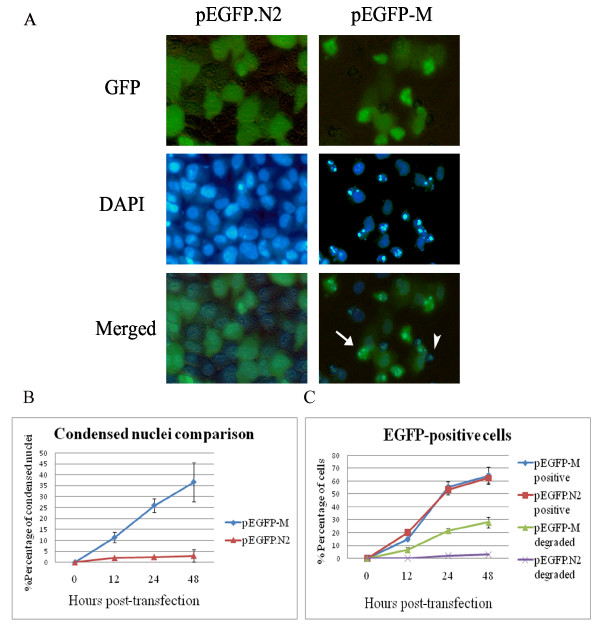
**Transient transfection of HeLa cells with pEGFP-M leads to apoptosis**. **A**. HeLa cells were transfected with either pEGFP or pEGFP-M construct. At 20 h post-transfection, cells were stained with DAPI and visualized by fluorescence microscopy. Arrow indicates a dead EGFP-M-positive cells. Arrow-head indicates a dead cell that may have lost its fluorescence due to possible degradation of EGFP-M protein. **B**. Percentage of condensed nuclei as compared to control. **C**. Percentage of EGFP-positive cells. Each data point represents three independent measurements, and the error bars indicate the standard error of the mean.

In addition to PI staining and flow cytometry analysis, we examined the nuclear morphology of transfected cells. AF2240-M cDNA was subcloned into the pEGFP.N2 vector. The resulting pEGFP-M construct or pEGFP.N2 control vector was transfected into HeLa cells and stained with DAPI (Figure 6). Transfection with pEGFP-M led to cell death as evidenced by the presence of numerous condensed and fragmented nuclei. On the other hand, in the pEGFP.N2 vector control transfected cells, the nuclei remained mostly intact.

**Figure 7:**
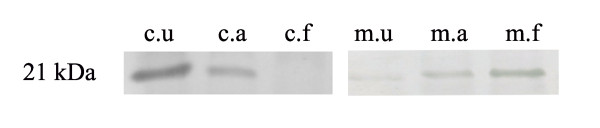
**Subcellular localization of Bax in pEGFP-M-transfected HeLa cells**. At 20 h post-transfection the protein samples were analyzed by Western blotting analysis with the anti-Bax 2D2 antibody. Transfected adherent cells were collected separately from transfected floating cells (c = cytosolic fraction, m = heavy membrane fraction, u = untransfected cells, a = transfected adherent cells, f = transfected floating cells). Bax translocated to the heavy membrane fraction after transfection with pEGFP-M, especially in transfected floating cells.

Moreover, in order to study the localization of Bax following transfection with pEGFP-M subcellular fractionation was carried out (Figure 7). Cytosolic and heavy membrane fractions containing mitochondria were subjected to Western blotting analysis with the anti-Bax 2D2 antibody. Bax was primarily localized to the cytosolic fraction before transfection. However, the Bax level was decreased in the cytosolic fraction following transfection with pEGFP-M. In contrast, the Bax level was increased in the heavy membrane fraction of transfected cells, especially that obtained from the floating cells.

### AF2240-M binds to human Bax

**Figure 8:**
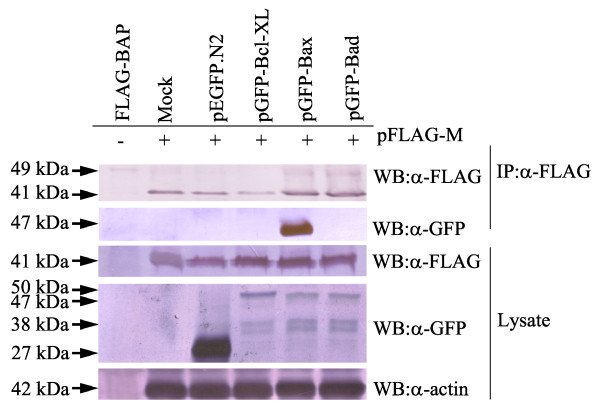
**AF2240-M interacts with Bax**. HeLa cells were co-transfected with pGFP-Bad, pGFP-Bax, pGFP-Bcl-XL or pEGFP.N2 and pFLAG-M, followed by lysis with 2% CHAPS buffer. Cell lysates were co-IPed with anti-FLAG M2-coated beads and Western blotted with either anti-FLAG M2 itself or anti-GFP. FLAG-BAP was used as control to evaluate successful IP. Total cell lysates were also directly Western blotted with either of the two antibodies. The double band at 38 kDa is non-specific. The amounts of total cell lysates loaded were verified by the actin.

HeLa cells were co-transfected with pGFP-Bax, pGFP-Bad or pGFP-Bcl-XL and pFLAG-M. At different time points post-transfection, cells were lysed in 2% CHAPS buffer as described above. Co-IP was carried out by incubation of the cell lysate with anti-FLAG M2-coated protein G agarose beads. Later, the samples were run on SDS-PAGE and subjected to two separate immunoblottings with anti-FLAG M2 and anti-GFP primary antibodies (Figure 8). Interestingly, GFP-Bax band was detected in anti-GFP treated blotting membranes, revealing that FLAG-tagged AF2240-M directly interacted with GFP-Bax. In addition, anti-FLAG M2 was able to label FLAG-tagged AF2240-M on the membrane, confirming successful purification of the protein by the resin. The GFP-Bad and GFP-Bcl-XL did not show such interaction with AF2240-M.

**Figure 9:**
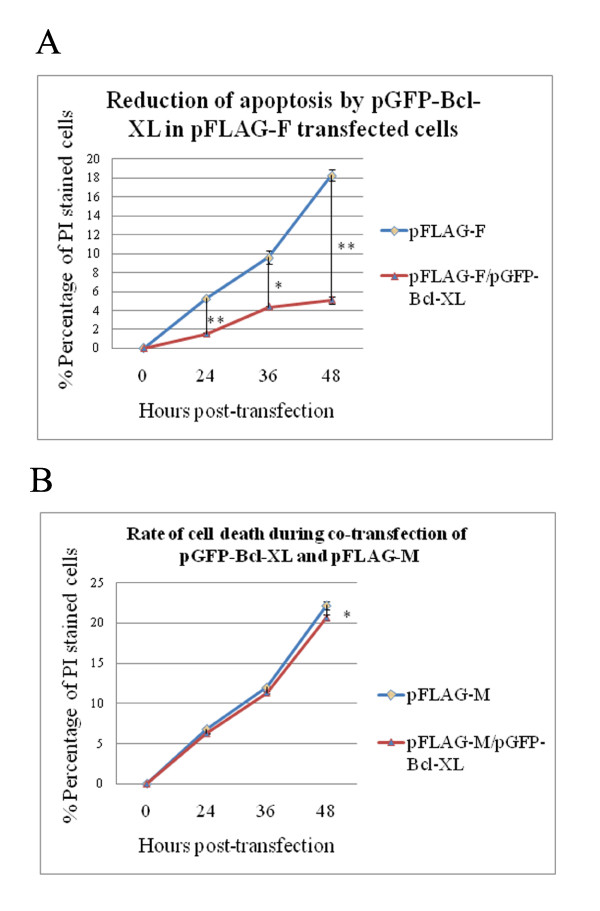
**Flow cytometry data after co-transfection of pFLAG-M or pFLAG-F with pGFP-Bcl-XL**. **A**. pGFP-Bcl-XL reduces the cell death caused by pFLAG-F by nearly five-fold. **B**. pGFP-Bcl-XL's effect on the rate of cell death caused by pFLAG-M was less significant. Data are obtained at different time points 24 h, 36 h and 48 h. Each data point represents three independent measurements, and the error bars indicate the standard error of the mean. *, p < 0.05 and **, p < 0.01 were referred to as statistically significant differences.

In addition, it was shown that the rate of cell death caused by pFLAG-F was decreased dramatically (five-fold) when it was co-transfected with equal amount of pGFP-Bcl-XL (Figure 9A). However, the co-IP experiments showed no interaction between GFP-Bax, GFP-Bad or GFP-Bcl-XL and FLAG-tagged AF2240-F. Moreover, in a parallel experiment, the cells were co-transfected with pcDNA3-FLAG-BimL and pEGFP-M, but no interaction was observed either (data not shown).

### AF2240-M interacts by its BH3 domain

**Figure 10:**
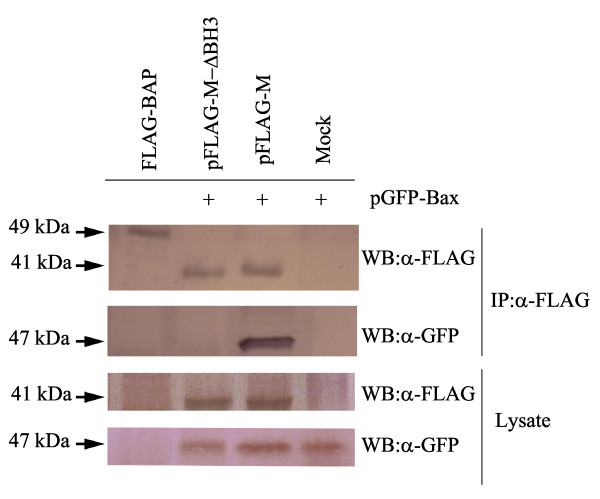
**AF2240-M-ΔBH3, unlike AF2240-M, does not interact with Bax**. HeLa cells were co-transfected with pFLAG-M or pFLAG-M-ΔBH3 and pGFP-Bax, followed by lysis with 2% CHAPS buffer. Cell lysates were co-IPed with anti-FLAG M2-coated beads and then Western blotted with either anti-FLAG M2 itself or anti-GFP. FLAG-BAP was used as control to evaluate successful IP. Total cell lysates were also directly Western blotted with either of the two antibodies.

In order to investigate if the BH3 domain in AF2240-M is responsible for binding to Bax, the truncated pFLAG-M-ΔBH3 was co-transfected with pGFP-Bax. After co-IP, although FLAG-tagged AF2240-M-ΔBH3 was present on the membrane blotted with anti-FLAG M2, no GFP-Bax was detected on the membranes blotted with anti-GFP (Figure 10). This showed that FLAG-tagged AF2240-M-ΔBH3 is not able to bind to GFP-Bax.

## Discussion

Our previous results did not show any significant changes in the total levels of Bax and Bcl-2 proteins following infection of HeLa cells with NDV [15]. In agreement with that, here we have shown that after infection with NDV, there was very little alteration on the mRNA levels of Bax and Bcl-2 from those levels (Figure 1). However, this event could also be strain and/or cell line-dependent; as shown previously [36], the Bax to Bcl-2 ratio in Vero cells following NDV infection changes. It has been observed that after infection of these cells with NDV, the Bax expression increased whereas Bcl-2 expression decreased. Therefore, it could be concluded that NDV-induced apoptosis in these two cell lines could follow different mechanisms.

Previously, we have shown that AF2240 causes cell death in HeLa cells within 24 h [15]. This event is associated with Bax conformational change and translocation from the cytoplasm to mitochondria. Subsequently cytochrome c is released from mitochondria and contributes to cell death via the intrinsic pathway of apoptosis. In the current study, we further investigated the reason behind pro-apoptotic ability of NDV by demonstrating that AF2240-M, AF2240-F and AF2240-L proteins contain BH domain-like regions (Figure 2). By cloning the AF2240-F and AF2240-M genes (that both contain BH1- and BH3-like domains) into expression vectors and transfecting them into HeLa cells, it was shown that the expressed proteins displayed pro-apoptotic effect (Figure 5). Later, we showed that mutant AF2240-M-ΔBH3 had nearly five-fold decrease in cell death rate compared to the wild-type, while AF2240-M-N-terminal had the same rate of cell death as to that of the full-length AF2240-M (Figure 5A).

Therefore, it was very likely that the BH domain-like regions are responsible for the apoptotic effect of NDV viral proteins and that they might interact with Bcl-2 family members. To further investigate, several plasmid constructs were co-transfected with another into HeLa cells. Based on co-IP, it was found that the FLAG-tagged AF2240-M indeed directly bound to the GFP-Bax protein (Figure 8). All other tested proteins of the Bcl-2 family failed to interact with either AF2240-M or AF2240-F. Furthermore, FLAG-tagged AF2240-M-ΔBH3 failed to interact with GFP-Bax proving that the binding occured via the BH3 domain of AF2240-M (Figure 10). It was concluded that the AF2240-M can also directly bind to human Bax following NDV infection as long as the plasmid constructs expressed proteins that resembled their natural forms.

Together with our previous results, it could be concluded that binding of the AF2240-M to the endogenous Bax results in activation of Bax and consequently contributes to its translocation to the outer membrane of mitochondria (Figure 7), facilitating the release of mitochondrial factors such as cytochrome c. It should be reminded that each NDV virus particle contains a large number of M protein molecules in the inner side of its membrane. After NDV entry, all viral proteins are released inside the host cell, and therefore each M protein molecule could potentially act as a killer agent to promote apoptosis. On the other hand, it has been reported that inactive NDV virus (either chemically fixed or UV-irradiated) can also exhibit pro-apoptotic ability [37,38]. Moreover, it has been shown that the substitutions of both HN and F proteins of mesogenic strains with velogenic strains did not alter nor increase the virulence of the virus [39], suggesting other factors are involved. These findings further supported our hypothesis that the M protein is a major candidate for the behavior of NDV.

As shown in Figure 5A, transfection of cells with pFLAG-F led to cell death, albeit somewhat lower compared to pFLAG-M. Surprisingly, co-transfection of pFLAG-F and the same amount of pGFP-Bcl-XL led to a dramatic five-fold drop of death rate (Figure 9A). This suggests a possible intracellular inhibition of AF2240-F by Bcl-XL. However, no interaction between FLAG-tagged AF2240-F and GFP-Bcl-XL was observed. Possibly, GFP-Bcl-XL inhibits other mechanisms downstream of the action of AF2240-F. In contrast, the reduction of apoptosis in pGFP-Bcl-XL and pFLAG-M co-transfected cells was not as significant as pGFP-Bcl-XL and pFLAG-F co-transfected cells (Figure 9B). Perhaps, AF2240-F and AF2240-M follow different pathways of apoptosis and/or GFP-Bcl-XL is not able to sufficiently prevent AF2240-M.

BH3-like region of AF2240-M is located within the N-terminal segment of the protein (a.a. 27-32). On the other hand and as stated before, the NDV M protein is believed to play an important role in the assembly of the virus by interacting with the nucleocapsid, the lipid bilayer and also the regions of the surface glycoproteins that are exposed on the inner surface of the membrane, but the domains on the M protein that are involved in binding with the three macromolecules have yet to be delineated [7]. Surprisingly, by using TMHMM software, we revealed that BH3-like region in AF2240-M protein is located in the only area that exhibits a minor transmembrane binding ability. Therefore, since many Bcl-2 family members interact with each other via their BH3 domain, further investigation is needed to show whether NDV M protein interacts with other NDV viral proteins via this domain.

It should be highlighted that although AF2240-L showed a higher sequence similarity to the BH3 domain compared to the rest of the viral proteins (Figure 2C), there is limitation for cloning and transfection studies because of the length of its gene (6615 bp).

On the other hand, a number of viral proteins have been identified that share homology with mammalian proteins and display either an anti- or pro-apoptotic effect upon introduction into host cells. We have tabulated some of these viral proteins that share a partial sequence similarity with Bcl-2 family members in Table 2. Many of these proteins contain Bcl-2 homology domains and interact with one or more Bcl-2 family members. For example, gamma-herpesviruses and few other herpesviruses encode at least one homolog of Bcl-2 [40]. African swine fever virus and Adenovirus encode at least two proteins with BH3 domains that mainly interact with pro-apoptotic members of Bcl-2 family [23,27,28,41]. However, most of these viral proteins show anti-apoptotic effect, and only few of them such as HCV core protein and HBV HBSP demonstrate pro-apoptotic effect. Here, we added NDV and its viral proteins to this group as not only they contain BH1- and BH3-like domains and display a pro-apoptotic property, but also AF2240-M interacts with Bax via its BH3 domain. Similar regions between Bcl-2 family and NDV proteins could have evolved during early evolution.

**Table 2:** Viral proteins that resemble Bcl-2 family members by having one or more BH-like domains.

Name	Viral Protein	Function	Interacts With	Does Not Interact With	Annotation	Reference
Vaccinia Virus	FL1	Anti-apoptotic	Bax, Bak, Bim	-	BH3 Domain	[29,45]
HCV	Core protein	Pro-apoptotic	Mcl-1	Bcl-XL, Bcl-w	BH3 Domain	[46]
Epstein-Barr Virus	BHRF1	Anti-apoptotic	Bax, Bak, Bim	-	BH3 Binding Groove	[21,22,47]
Gamma-Herpes Virus 68	M11, vBcl-2	Anti-apoptotic	Bim	-	-	[40,48]
Myxoma Virus	M11L	Anti-apoptotic	Bak	-	BH3 Domain	[49]
African Swine Virus	A179L, 5-HL	Anti-apoptotic	Bid, Bax, Bak, Noxa	-	BH1 Domain	[23,41]
Kaposi Sarcoma-Associated Virus	ORF K12	Anti-apoptotic	-	Bak, Bax	Human Herpesvirus-8. 9 Homologs	[24,25,50]
HBV	HBSP	Pro-apoptotic	-	-	BH3 Domain	[51]
Herpesvirus Saimiri	ORF16	Anti-apoptotic	Bax, Bak	-	Conserved BH1, BH2. No BH3	[26,52]
Orf Virus	ORFV125	Anti-apoptotic	Bik, Puma, Noxa, Bim	Inactive form of Bax	BH3 Domain	[53]
Adenovirus	E1B-19K	Anti-apoptotic,	Bax	Bcl-2	BH3 Domain	[28]

So far only few viral proteins discovered show pro-apoptotic characteristics compared to the rest with anti-apoptotic effects.

The BH3 domain is mainly considered as a pro-apoptotic domain of the Bcl-2 family of proteins [42]. Although the BH1 domain is generally a pro-survival domain, it is present in some of the pro-apoptotic members such as Bak and Bax. In this study, we show that although AF2240-M and AF2240-F proteins both contain BH1-like regions, they both display a pro-apoptotic function (Figure 5). However, BH1-like domain of AF2240-M was not found to be responsible for its interaction with Bax.

Not much is known about the mechanisms underlying the velogenicity of different strains of NDV, but there is now evidence that the cleavability of F0 is a major determinant for virulence of NDV virus [43,44]. Therefore, some might argue that a direct transfection of a plasmid containing full-length F gene would not fully demonstrate its pro-apoptotic ability as the expressed product has not undergone post-translational modification, and therefore it is not in its natural wild-type form. However, we noticed that the BH1- and BH3-homologous regions of AF2240-F are not localised within any of the previously characterised regions (*i.e*. HR regions, cleavage site, glycosylation regions, or transmembrane segment). Thus, AF2240-F could still exert its apoptotic effect whether it is in its full-length or cleaved form. On the other hand, unlike F and HN glycoproteins of NDV, the M protein does not undergo cleavage and glycosylation. There has been no report showing that M core protein has a major post-translational cleavage similar to that of NDV surface proteins. Therefore, transfection of a full-length M gene and elicitation of its pro-apoptotic effect would unlikely to be regulated by post-translational modification.

AF2240 is a velogenic strain that causes severe intestinal lesions and neurological disorders, resulting in high mortality. Previously it was shown that AF2240-HN protein of this strain has a different length compared to those of other strains [30]. Compared to other NDV strains, the BH3 domain-like sequence in AF2240-M has threonine 38 residue in place of alanine (Figure 3). In addition, glycine 2130 is present in the BH3 domain-like sequence of AF2240-L protein in place of serine. On the other hand, most L proteins share residues that are not present in AF2240-L. These differences could potentially account for the velogenic characteristic of AF2240 compared to milder strains and this is in need of further investigation.

In conclusion, we have demonstrated that the NDV M protein is able to interact with human Bax protein by its BH3 domain. Such interactions could potentially account for the pro-apoptotic ability of NDV and ultimately lead us to better understanding of the viral mechanisms.

## Abbreviations

ATCC: American type culture collection; DMEM: Dulbecco's modified Eagle's medium; FBS: Fetal bovine serum; BH domain: Bcl-2 homology domain; Ct: Cycle threshold.

## Competing interests

The authors declare that they have no competing interests.

## Authors' contributions

AM designed and performed the experiments and drafted the manuscript. YTH participated in the design of the study, performed the data analysis and contributed to writing the manuscript. FJ assisted in the alignment studies. SA participated in the data analysis. RR conceived of the study and participated in its coordination. KY participated in the design of the study, financial support and contributed to writing the manuscript. All authors have read and approved the final manuscript.
